# *Phellodendron amurense* Extract Protects Human Keratinocytes from PM2.5-Induced Inflammation via PAR-2 Signaling

**DOI:** 10.3390/biom11010023

**Published:** 2020-12-28

**Authors:** Jiyoung Choi, Mi Yeon Moon, Gi Yeon Han, Moon Sik Chang, Dongki Yang, Joonseok Cha

**Affiliations:** 1 Research Center, The Garden of Naturalsolution, Gyeonggi-do 18103, Korea; jychoi@naturalsolution.co.kr (J.C.); mares1982@naturalsolution.co.kr (M.Y.M.); elin_han@naturalsolution.co.kr (G.Y.H.); justin0510@naturalsolution.co.kr (M.S.C.); 2 Department of Physiology, College of Medicine, Gachon University, Incheon 21999, Korea

**Keywords:** skin inflammation, particulate matter (PM), proteinase-activated receptor-2 (PAR-2)

## Abstract

Dietary supplement and personal care products aiming to provide protection from air pollution have been of great interest for decades. Epidemiology demonstrated that PM10 and PM2.5 particulate matter (PM) are an actual threat to public health worldwide, but the detailed processes of how these particles attack the cells are not fully understood. Here, we report that the measurement of intracellular calcium concentration ([Ca^2+^]*i*) using human respiratory or skin cells can illustrate pollutant challenges by triggering Ca^2+^ influx in these cells. This signal was generated by proteinase-activated receptor-2 (PAR-2), confirmed by competition analyses, and *Phellodendron amurense* bark extract (PAE), a traditional medicine, was able to control the response and expression of PAR-2. Increase in proinflammatory cytokines and decrease in cell adhesion components could suggest a severe damage status by air pollutants and protection by PAE. Finally, we identified 4-O-feruloylquinic acid (FQA), an active compound of PAE, showing the same effects on Ca^2+^ influx and PAR-2 regulation. The results presented here should help understand the underlying mechanism of PM insults and the beneficial effect of standardized PAE as dietary supplement or cosmetical ingredient.

## 1. Introduction

Air pollution is a major environmental threat to public health worldwide [1,2]. The World Health Organization (WHO, Geneva, Switzerland) Air Quality Guidelines confirmed that over 4 million premature deaths were probably caused by particulate matter (PM)2.5 [3]. It is widely accepted that PM10 and PM2.5, smaller than 10 and 2.5 μm respectively, are generated mostly from the use of fossil fuels and cause diseases and cancers related to cardiovascular and respiratory systems [4]. Several researchers have concluded that outdoor or ambient air pollution is carcinogenic to humans, and, more recently, large-scale retrospective analyses of over 600 cities worldwide revealed an independent association between short-term exposure to air pollutants and daily mortality [5,6,7].

Other than respiratory tracts, skin is an important organ that is in constant contact with air pollution. Skin is the biggest and outmost organ in our body, and it plays a role as a barrier to environmental insults, both physical and chemical [8]. The stratum corneum, at the top of the skin surface, is challenged on many external stresses, including reactive oxygen species (ROS) produced by particulate matters, and often PM2.5 can penetrate into deeper layers of the skin, resulting in skin aging symptoms [9]. Exposure to air pollutants also causes premature skin aging, pigmentation spots, and acne. Like airway mucosa, human dermal fibroblasts and immortalized nontumorigenic epithelial (HaCaT) cells have been shown to struggle with inflammatory conditions elicited by air pollutants [10,11,12,13]. Cumulative irritations by exposure to PM have been implicated in skin disorder statuses, such as psoriasis, atopic dermatitis, and even skin cancer. Studies have suggested that proinflammatory cytokines and the NF-κB pathway indicate damage status by air pollutants, probably with ROS also being generated by them. Pollutants can also disrupt the integrity of skin barrier function, facilitating further penetration through the epidermal layers and leading to the complex toxicity. Still, it remains unclear which signal transduction is induced by the contact with PM and activates the downstream pathways to determine cellular responses, especially inflammatory conditions.

Intracellular calcium (Ca^2+^) is a universal intracellular second messenger, mostly implicated in muscle contractile activation [14]. The level of cytosolic Ca^2+^ concentration is critically regulated by living cells, maintained much lower than the extracellular environment. Plenty of energy is consumed by Ca^2+^ pumps, and highly regulated intracellular compartments (Ca^2+^ stores) are also important for cell viability [15]. Immediate influx of cytosolic Ca^2+^ indicates that the cells sense environmental changes via various surface molecules, and a wide range of Ca^2+^-dependent regulators have been studied by monitoring intracellular calcium concentration ([Ca^2+^]*i*). In 2011, Li et al., reported that the contact of human bronchial epithelia to diesel exhaust particles induced Ca^2+^ influx [16]. Proteinase-activated receptor-2 (PAR-2) was involved in the activation of transient receptor potential vanilloid, family member 4 (TRPV4), leading to the disease symptoms of respiratory cells. It is interesting to note that PAR-2 has been known as a critical player in chronic inflammation by physiological and molecular researchers [17].

Using traditional medicines or dietary compounds to enhance skin health has seen an increasing demand in recent decades. Cosmeceutical products and supplements are leading the personal care trend, and applying biomolecules from medicinal plants is widely reported. In this study, we established stable platforms that can monitor [Ca^2+^]*i* during respiratory or skin cells in contact with PM2.5 and respond to the stress. Transient, but consistent Ca^2+^ influx was observed upon the diesel particle matter (DPM) treatment in both normal human bronchial/tracheal smooth muscle (HBT-SM) and HaCaT cells. The involvement of PAR-2 was confirmed by competition analyses with a synthetic agonist and an antagonist. Furthermore, we evaluated a traditional medicine, *Phellodendron amurense* bark extract (PAE), to inhibit PM-induced Ca^2+^ influx by directly acting on PAR-2, alleviate inflammation and maintain homeostatic levels of cell adhesion components. Our study uncovered hidden signal transduction that cells use to rapidly respond to the external pollutants. These findings also elucidate the mechanism of PAE’s cosmeceutical effect, which was recently suggested in an atopic dermatitis research [18].

## 2. Materials and Methods

### 2.1. Treatment of the Cells with DPM, PAE, and 4-O-Feruloylquinic Acid (FQA)

DPM NIST^®^ SRM^®^ 1650b, Fura-2/AM and Pluronic™ F-127 were purchased from Merck and Invitrogen. DPM stock solution (25 mg/mL) was prepared in dimethyl sulfoxide (DMSO) and sonicated in a water bath with 40% amplitude and for 30 s on/off for 3 cycles. Because the particles are not solubilized, we kept the same protocol to prepare the suspension directly before treating the cells.

The doses of PAE and FQA were determined after testing the cytotoxicity of each, as described in Supplementary Materials Figure S1.

### 2.2. Measurement of [Ca^2+^]i

HaCaT cells were kindly provided by the Department of Genetic Engineering, College of Life Science and Skin Biotechnology Center, Kyung Hee University, and HBT-SM cells were purchased from LIFELINE Cell Technology (Frederick, MD, USA). The cells were plated at a density of 50,000 cells/well per coverslip, placed in a 12-well plate 15 h before the experiment, and grown in Dulbecco’s modified Eagle’s medium (DMEM). Cells were loaded with 5 μM Fura-2/AM (Invitrogen, Carlsbad, CA, USA) and 0.1% Pluronic™ F-127 (Invitrogen) and incubated at 37 °C for 30 min in a bath solution (10 mM HEPES (pH 7.4), 140 mM NaCl, 1 mM MgCl_2_, 5 mM KCl, 2 mM CaCl_2_, and 10 mM glucose). Cells attached to the coverslips were perfused with the prewarmed (37 °C) bath solution at the bottom of a perfusion chamber. DPM and the other specimens were diluted in the bath solution. [Ca^2+^]*i* was measured by using 340 and 380 nm excitation, and the emitted fluorescence was captured by digital CCD camera (Hamamatsu Photonics, Hamamatsu, Japan) at 510 nm to be analyzed by Metafluor software (Molecular Devices, San Jose, CA, USA). A fluorescence ratio of 340/380 nm was recorded, and the responses to the specimens were calculated as the percentage of changes in fluorescence compared to each control. Mean ± SE of the percentage values from individual cells were calculated and plotted with the number of cells measured. The same amount of extraction vehicle (butylene glycol for PAE, or solvent DMSO for FQA) was added to the control.

### 2.3. Quantitative Real-Time PCR

HaCaT cells were plated at a density of 3 × 10^4^ cells per well in a 96-well plate and cultured in a 5% CO_2_ incubator at 37 °C for 24 h. Then, DPM (25 μg/mL) and PAE or FQA were treated with the cells and incubated for 37 °C for 24 h. The same amount of extraction vehicle (butylene glycol for PAE, or solvent DMSO for FQA) was added to the control. The cells were washed twice with PBS, and preparation of cell lysates and reverse transcription were performed using a commercial kit (SuperPrep II Cell Lysis & RT Kit for qPCR; TOYOBO, Osaka, Japan). The concentration of the extracted cDNA was measured using a NanoDrop (Nanodrop 2000, Thermo Fisher Science, Waltham, MA, USA). To quantitate the expression levels of the target genes, quantitative RT-PCR was performed on the LightCycler^®^ 96 (Roche Diagnostic, Mannheim, Germany) using a FastStart Essential DNA Green Master (Roche Diagnostic, Mannheim, Germany) with the primers in Table 1. The gene expressions were compared for analysis by normalizing against β-actin expression.

### 2.4. Western Blot Analysis

HaCaT cells were plated at a density of 5 × 10^5^ cells/well in a 6-well plate and cultured in a 5% CO_2_ incubator at 37 °C for 24 h. Then, DPM (25 μg/mL) and PAE or FQA were treated with cells and incubated for another 24 h. The same amount of extraction vehicle (butylene glycol for PAE, or solvent DMSO for FQA) was added to the control. The cells were washed twice with cold PBS and lysed with RIPA buffer containing protease inhibitor cocktail. The lysates were centrifuged at 4 °C, 13,000 rpm for 15 min. Supernatants were harvested, and protein quantification was performed by Bradford analysis. Samples with equal amounts of protein (20 μg) were separated using 4–12% SDS polyacrylamide gels, transferred to polyvinylidene difluoride membranes (PVDF), and blocked with 5% BSA in TBST buffer for 25 °C for 1 h. Antibody dilutions were as follows: PAR-2, 1:1000; occludin, 1:1000; ZO-1, 1:1000; β-actin, 1:10,000. Antibodies specific for PAR-2 (#6976), occludin (#91131), and ZO-1 (#13663) were purchased from Cell Signaling Technology (Beverly, MA, USA). The antibody for β-actin (A1978) was from Sigma Aldrich (St. Louis, MO, USA). Detection of bands was performed using Image Quant LAS 4000 (GE Healthcare, IL, USA) hardware and software, according to the manufacturer’s instructions. Signal intensities were quantified by densitometry, with Image J (NIH, Bethesda, MD, USA).

### 2.5. Isolation of Active Compounds in PAE

The bark of *P. amurense* was extracted with 50% ethanol, and PAE was sequentially fractionated with hexane, chloroform, ethyl acetate, and butanol. Three known compounds, namely, palmatine, berberine, and 4-O-feruloylquinic acid (FQA) were isolated from the ethyl acetate and the butanol fractions. The structure of each isolate was determined with NMR spectroscopy.

Palmatine: ^13^C-NMR (100MHz, CD_3_OD) δ 110.4 (C-1), 123.7 (C-1a), 151.3 (C-2), 154.3 (C-3), 112.7 (C-4), 130.5 (C-4a), 28.3 (C-5), 57.1 (C-6), 146.9 (C-8), 120.9 (C-8a), 146.2 (C-9), 152.3 (C-10), 124.9 (C-11), 128.5 (C-12), 135.7 (C-12a), 121.7 (C-13), 140.2 (C-13a), 58.1 (C-2, OCH_3_), 57.4 (C-3, OCH_3_), 63.0 (C-9, OCH_3_), 57.8 (C-10, OCH_3_) [24].

Berberine: ^13^C-NMR (100MHz, CD_3_OD) δ 106.6 (C-1), 121.9 (C-1a), 150.0 (C-2), 152.1 (C-3), 109.5 (C-4), 132.0 (C-4a), 28.3 (C-5), 57.7 (C-6), 146.5 (C-8), 123.4 (C-8a), 145.8 (C-9), 152.2 (C-10), 128.1 (C-11), 124.6 (C-12), 135.2 (C-12a), 121.6 (C-13), 139.7 (C-13a), 58.1 (C-2, OCH_3_), 57.4 (C-3, OCH_3_), 62.6 (C-9, OCH_3_), 57.7 (C-10, OCH_3_) [25].

4-O-Feruloylquinic acid: ^13^C-NMR (100MHz, CD_3_OD) δ 74.1 (C-1), 38.3 (C-2), 72.5 (C-3), 79.0 (C-4), 72.3 (C-5), 40.1 (C-6), 181.4 (C-7), 127.8 (C-1′), 111.8 (C-2′), 147.3 (C-3′), 149.5 (C-4′), 116.4 (C-5′), 124.2 (C-6′), 149.0 (C-7′), 115.5 (C-8′), 169.8 (C-9′), 57.4 (C-3, OCH_3_) [26].

### 2.6. Statistical Analysis

Data of all experiments are expressed as mean ± SEM. Significant differences (* *p* < 0.05, ** *p* < 0.01, and *** *p* < 0.001) between the control and each experimental group from more than 3 independent repeats were confirmed by one-way ANOVA or paired student’s *t*-test.

## 3. Results

### 3.1. Identification of a Pollutant-Induced Intracellular Signal

To characterize cellular damages elicited by air pollutants, especially PM2.5, we set up an intracellular Ca^2+^-measuring system as described in the Materials and Methods Section. We focused on [Ca^2+^]*i* because of its wide involvement in sensing environmental stimuli, and HBT-SM cells were chosen as a primary reporter because they are likely to be among the first to come into contact with air pollutants in the human body, and they are a favored cell model to study Ca^2+^ signaling.

**Figure 1:**
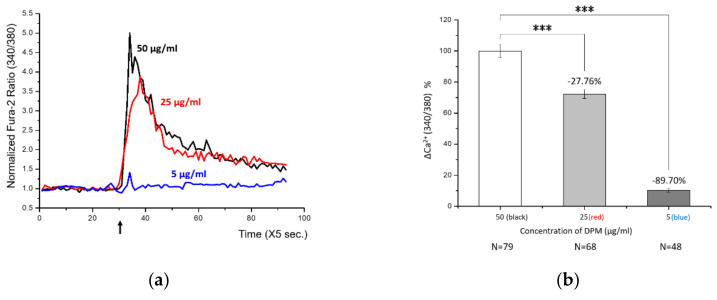
Diesel particle matter (DPM) induced transient Ca^2+^ influx in human bronchial/tracheal smooth muscle (HBT-SM) cells. (**a**) HBT-SM cells were treated with different concentrations of DPM at the indicated time (arrow), and intracellular calcium (Ca^2+^) concentration was monitored. Black, 50 μg/mL; red, 25 μg/mL; blue, 5 μg/mL. (**b**) Highly significant Ca^2+^ influx by DPM was observed in a concentration-dependent manner. N indicates the number of cells analyzed. (*** *p* < 0.001).

The experiment showed how the [Ca^2+^]*i* changes over time. After stabilization of HBT-SM cells in the current of media, we challenged cells with DPM. DPM was able to induce a transient increase in [Ca^2+^]*i*, which was soon reduced back to a level close to the original (Figure 1a). We interpret this decrease as a result of regulatory machineries maintaining the homeostasis. Different concentrations of DPM can lead to distinct responses: 50 μg/mL and 25 μg/mL showed similar but concentration-dependent levels and durations of Ca^2+^ influx, whereas 5 μg/mL showed only a marginal change which was barely noticeable by integration of the plot (Figure 1b).

### 3.2. Control of DPM-Induced Ca^2+^ Influx by PAE

**Figure 2:**
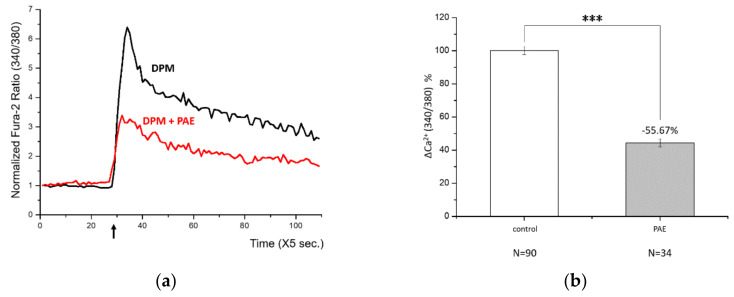

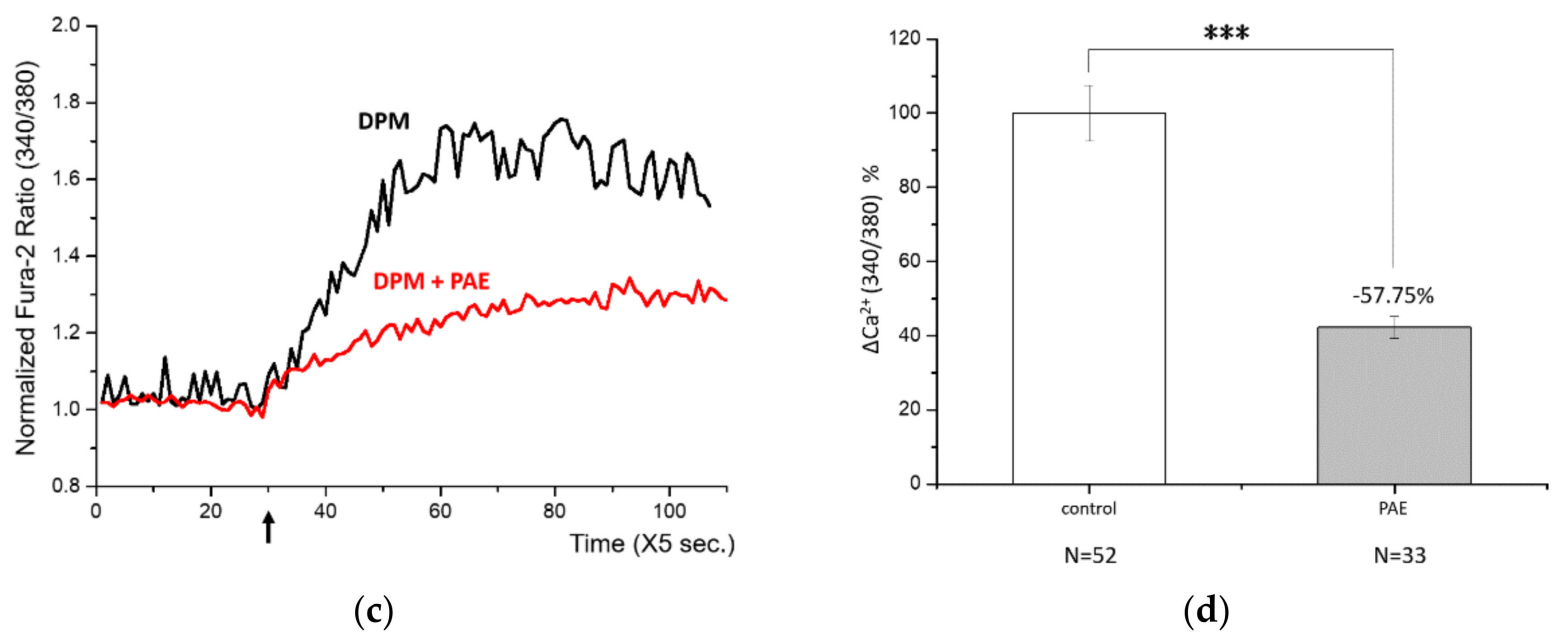
*Phellodendron amurense* bark extract (PAE) was able to reduce DPM-induced Ca^2+^ influx in both HBT-SM and immortalized nontumorigenic epithelial (HaCaT) cells. (**a**) HBT-SM cells were preincubated with PAE (blue and red), or not (black), and 25 μg/mL of DPM was treated at the indicated time (arrow). Blue and red represent independent sets of experiment. (**b**) The inhibition of Ca^2+^ influx by PAE was highly significant. (**c**) The same experiment was conducted using HaCaT cells. Red, preincubated with PAE; black, not. (**d**) DPM induced similar Ca^2+^ influx, which was inhibited by PAE, and the effect was also robust and significant in HaCaT cells. (*** *p* < 0.001).

After identifying a damage signal by DPM, we screened natural products to find potential candidates protecting from air pollution. Among many herbal remedies, PAE demonstrated consistent and significant inhibition of Ca^2+^ influx (Figure 2a). Rapid Ca^2+^ entry was reduced by more than half with high statistical significance (Figure 2b). We also tested the effect of PAE in the keratinocytes, which constitute human skin and are also among the first cells to come into contact with air pollutants. Interestingly, we observed similar Ca^2+^ influx by DPM and obvious inhibition by PAE in HaCaT cells (Figure 2c). Although the response curves look different in HBT-SM and HaCaT cells, reflecting distinct Ca^2+^ metabolism, PAE was able to consistently regulate the initial influx in both types of cells (Figure 2d).

### 3.3. PAR-2: A Mediator of DPM-Induced Ca^2+^ Influx

**Figure 3:**
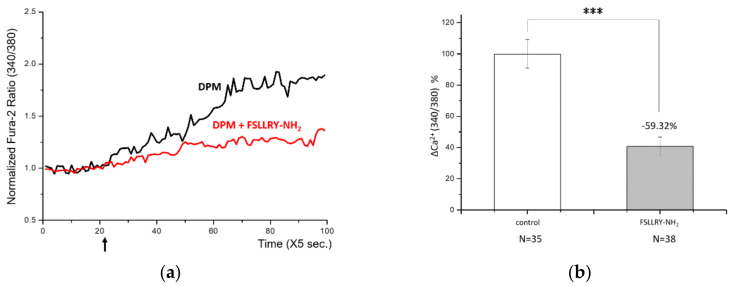

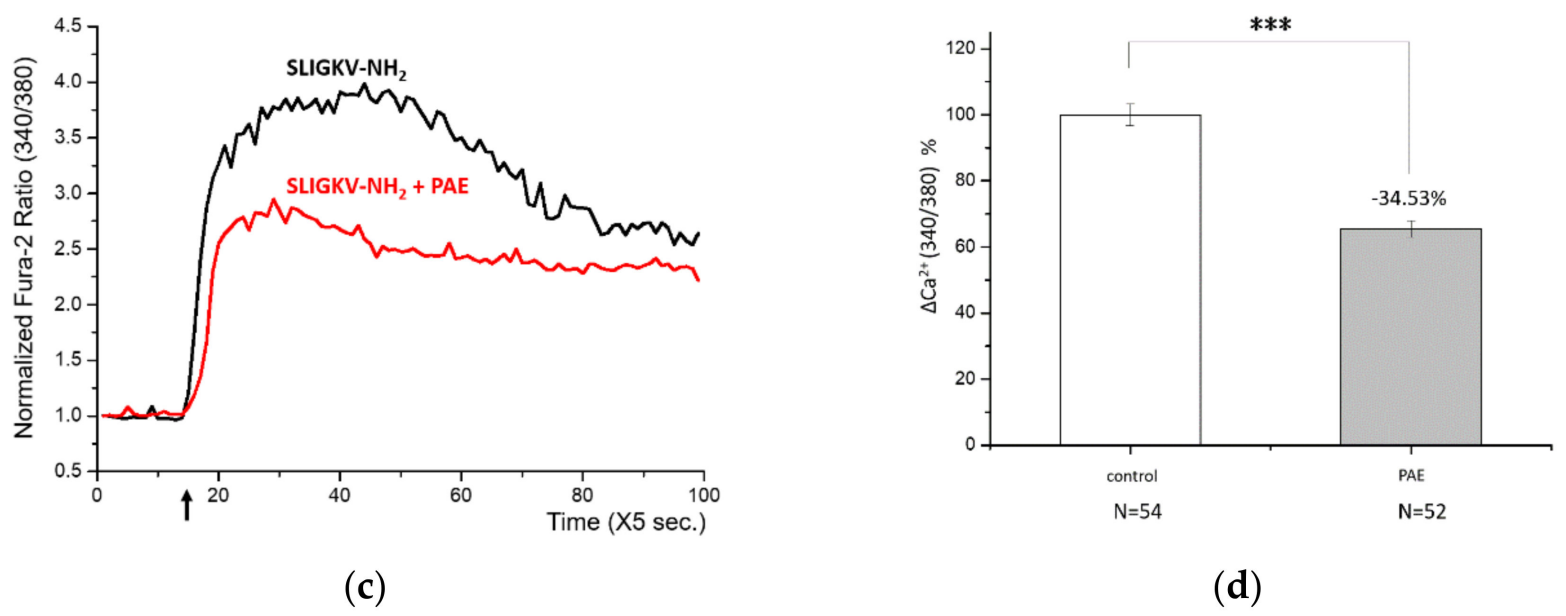
Proteinase-activated receptor-2 (PAR-2) was responsible for the DPM-induced Ca^2+^ influx. (**a**) HaCaT cells were treated with DPM only (black) or DPM and 100 μM FSLLRY-NH2 (red) at the indicated time (arrow). (**b**) DPM-induced Ca^2+^ influx was successfully reduced by inhibiting PAR-2. (**c**) HaCaT cells were treated with 100 μM SLIGKV-NH2 only (black) or SLIGKV-NH2 and PAE (red) at the indicated time (arrow). (**d**) The PAR-2 agonist induced Ca^2+^ influx, which was also inhibited by PAE. This result indicates that the effect of PAE was mediated by PAR-2 regulation. (*** *p* < 0.001).

Ca^2+^ signaling upon DPM treatment reminded us of the activation of PAR-2 by matrix metalloproteinase-1 (MMP-1) in human bronchial epithelia [16]. PAR-2 has been reported to activate TRPV4 channels and cause Ca^2+^ influx as we observed. This led us to check the responsibility of PAR-2 for the results we obtained, and a known synthetic peptide antagonist, FSLLRY-NH2, was employed [27]. HaCaT cells were treated with DPM with or without the antagonist, and DPM-induced Ca^2+^ influx was decreased by more than 50% by the antagonist (Figure 3a,b). This result demonstrates that PAR-2 is responsible for the calcium signaling of DPM.

Next, we treated HaCaT cells with a known PAR-2 agonist, SLIGKV-NH2, and it induced Ca^2+^ influx (Figure 3c,d), correlating with the known effect. When we treated cells with PAE at the same time, the calcium signaling by PAR-2 was significantly reduced while, the agonist constitutively activated PAR-2. Together with the results above, we can conclude that DPM activated PAR-2 and induced Ca^2+^ signaling, which was suppressed by PAE. It is likely that the regulation of PAR-2 was a direct action of PAE, given that PAE was able to counteract the PAR-2 agonist, albeit partly.

### 3.4. Regulation of PAR-2 Expression

**Figure 4:**
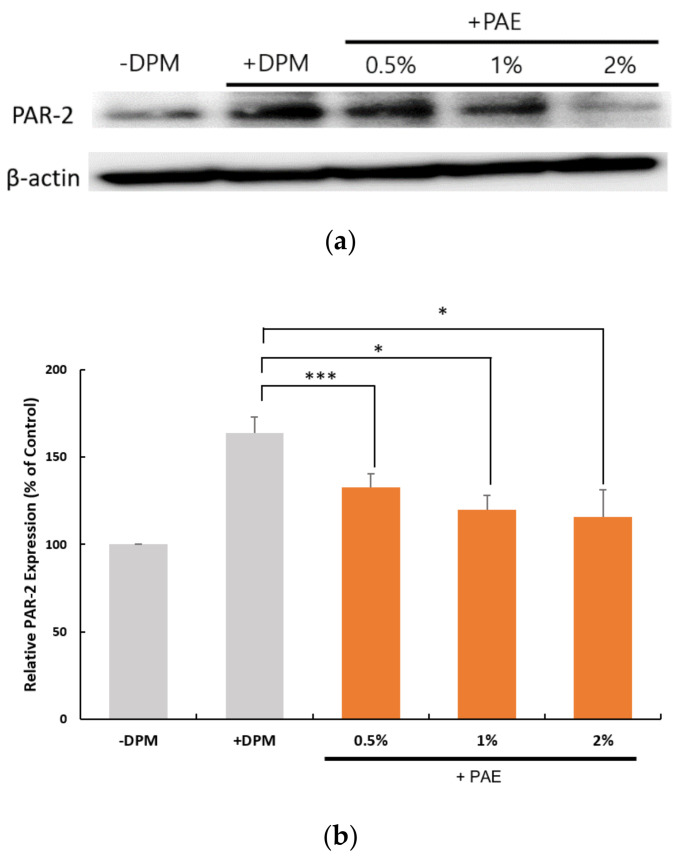
DPM and PAE modulated PAR-2 expression in opposite ways. (**a**) Western blot showed a robust increase in the PAR-2 protein caused by DPM in HaCaT cells, and the treatment with PAE was able to reverse the effect of DPM in a concentration-dependent manner. (**b**) Transcriptional regulation of PAR-2 was demonstrated to be upregulated by DPM, which could be inhibited by PAE. (* *p* < 0.05, *** *p* < 0.001).

To learn more about the role of PAR-2 in DPM-induced signaling, we monitored the expression of PAR-2 upon the treatment of DPM and the presence of PAE. As shown in Figure 4, the expression of PAR-2 was upregulated at both the protein and the mRNA levels, suggesting that the cells require PAR-2 to properly respond to the air pollutants. PAE, however, was able to reduce the levels of PAR-2 in a concentration-dependent manner, which reversed the change by DPM. This confirmed the protective activity of PAE against the air pollutants.

### 3.5. Inflammatory Conditions as an Outcome of DPM Challenge

As previously mentioned, PAR-2 has been known to function in diverse physiological conditions in different tissues [27]. PAR-2 mediates inflammatory responses in bronchial epithelia, which involves the regulation of interleukin-8 (IL-8) and interleukin-6 (IL-6) [28]. IL-8, IL-6, and tumor necrosis factor-α (TNF-α) have also been involved in a range of research of inflammatory conditions of human skin [11].

**Figure 5:**
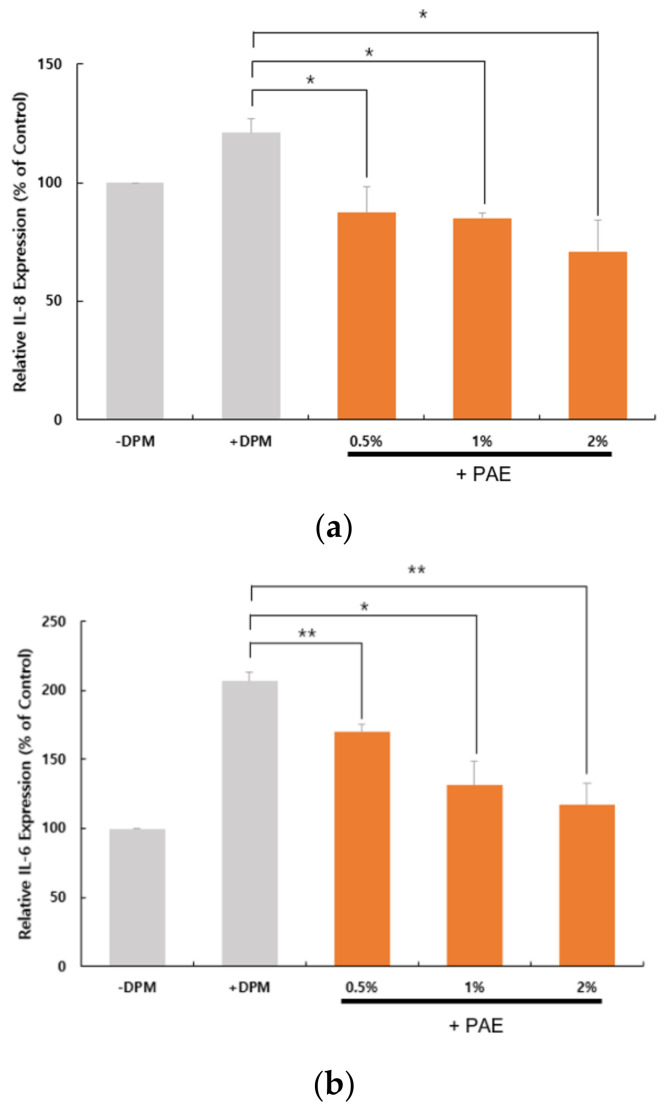

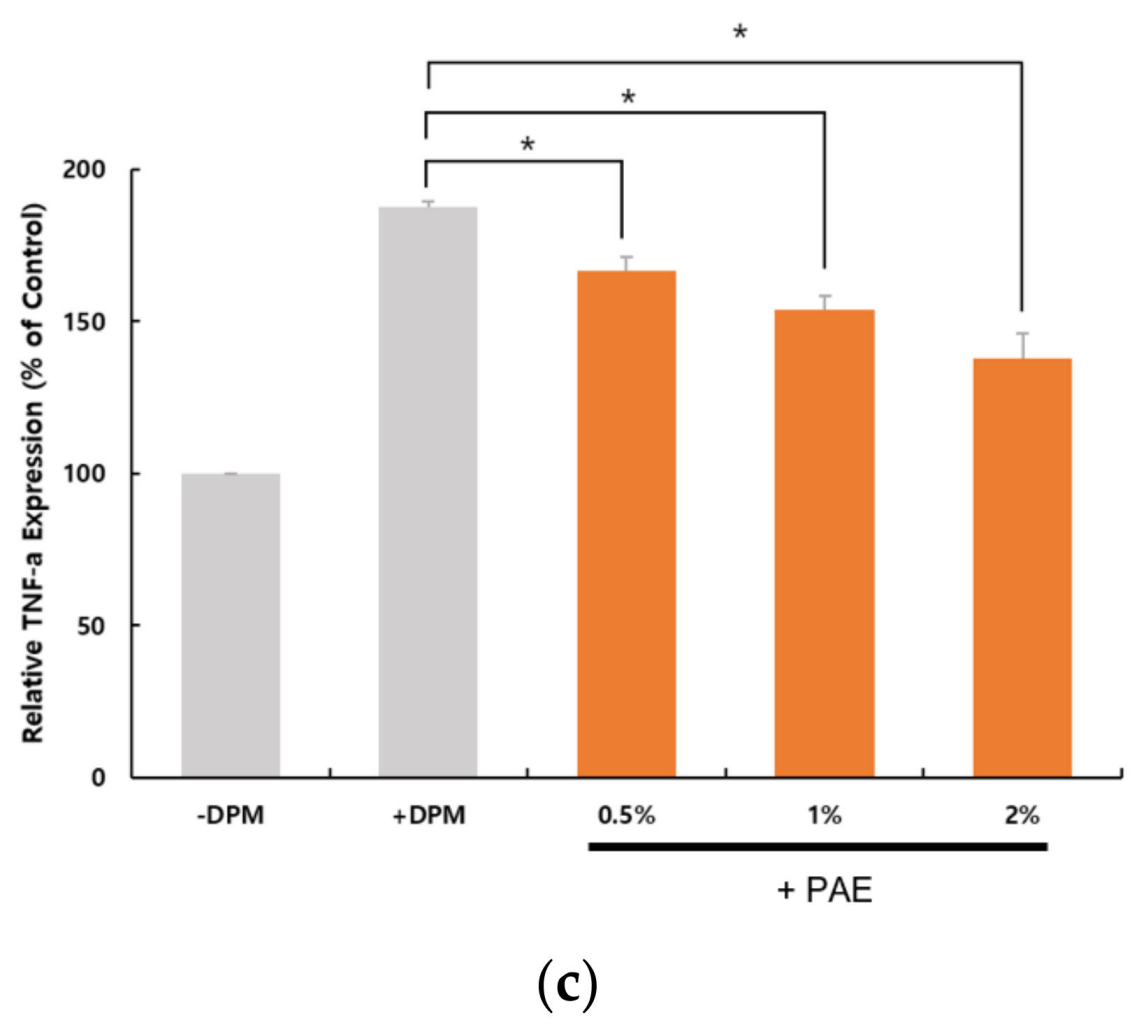
DPM upregulated proinflammatory cytokines and tumor necrosis factor-α (TNF-α). HaCaT cells were treated with DPM only or DPM and PAE. Transcriptional regulation of interleukin-8 (IL-8) (**a**), IL-6 (**b**) and TNF-α (**c**) indicates that DPM induces inflammatory responses, which could be inhibited by PAE in a concentration-dependent manner. (* *p* < 0.05, ** *p* < 0.01).

We examined the expression of proinflammatory cytokines to determine whether DPM-induced PAR-2 activation could lead to inflammatory conditions in HaCaT cells. At the transcriptional level, we were able to confirm that a set of proinflammatory cytokine genes, IL-8, IL-6 and TNF-α, were upregulated by the DPM treatment (Figure 5). Similar to the PAR-2 regulation, PAE inhibited the induction by DPM of the cytokines in a concentration-dependent manner. Because DPM activated PAR-2 for Ca^2+^ influx, these results are highly correlated with the previous observation that PAR-2 activates inflammatory pathways.

### 3.6. Skin Barrier Weakened by DPM and Protected by PAE

**Figure 6:**
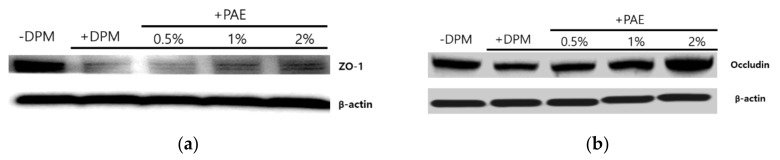
DPM dysregulated cell adhesion proteins, and PAE played a role in maintaining homeostasis. DPM displayed downregulation of desmosomal or tight-junction components. Zonula Occludens-1 (ZO-1) (**a**) and Occludin (**b**) expressions were repressed at the protein level, and the perturbation of homeostasis was reversed by PAE, indicating a protective role.

Skin inflammation is often accompanied with skin barrier dysregulation, resulting in various symptoms, e.g., atopic dermatitis or psoriasis [29,30]. Moreover, several studies demonstrated the association of PAR-2 activity with skin health, especially its barrier function [31]. To better understand the skin disorder conditions that DPM may be capable of inducing, we also monitored cell adhesion molecules in HaCaT cells, implicated in the skin barrier integrity. Among desmosomal and tight-junction components, we investigated the protein levels of Zonula Occludens-1 (ZO-1) and Occludin. Western blot analyses displayed consistent reduction of ZO-1 and Occludin upon DPM treatment, indicating potential skin barrier damage (Figure 6). The downregulation of these proteins was reversed by increasing concentrations of PAE, suggesting that PAE could also protect skin barrier integrity from air pollutants.

### 3.7. FQA: A Novel Antipollution Agent

From the results above, PAE was successfully characterized as an active ingredient that can protect HaCaT cells from a set of damages by air pollutants. We then tried to identify a single compound most responsible for this efficacy. We were able to isolate FQA and the other two known compounds through a series of fractionation followed by NMR spectroscopy, as described in the Materials and Methods Section (Figure S5).

**Figure 7:**
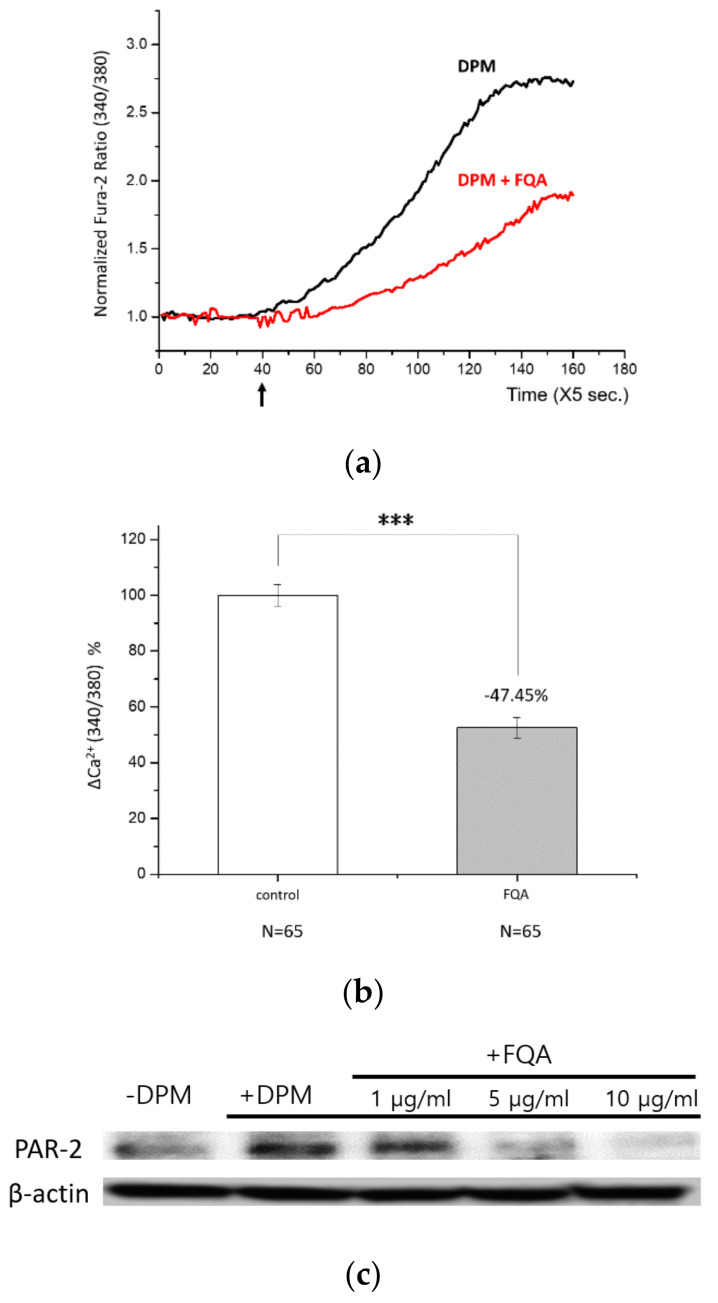
4-O-feruloylquinic acid (FQA) is a major determinant of protective function of PAE. (**a**) HaCaT cells were treated with DPM only (black) or DPM and 100 ppm FQA (red) at the indicated time (arrow). (**b**) DPM-induced Ca^2+^ influx was significantly reduced by FQA, hardly distinguishable from the effect of PAE. (**c**) The regulation of PAR-2 upon the DPM treatment was countered by FQA in a concentration-dependent manner. (*** *p* < 0.001).

First, we examined the Ca^2+^ influx by DPM in the presence of FQA. As shown in Figure 7a,b, FQA perfectly reproduced the protecting effect of PAE in HaCaT cells. The effect was concentration dependent, and an amount of 50 ppm was able to inhibit the influx by half to 100 ppm (data not shown). Second, the expression of PAR-2 was investigated. By the increasing amount of FQA, upregulation of PAR-2 by DPM was reversed to the normal status (Figure 7c). This change in PAR-2 level was also confirmed at the mRNA level (Figure S7). With these results, we can conclude that FQA is a major determinant of PAE’s effect on DPM-induced damages, and standardized natural extracts can be used as a dietary supplement or cosmetic ingredient to protect from air pollution.

## 4. Discussion

Air pollution has been one of the greatest public health concerns for decades, but many questions are still unanswered, and there remains a lack of proper research in vitro and in vivo. We started this study by asking what kind of signal can be generated by air pollutants, leading to the damage conditions at the cellular level. Ca^2+^ is an important second messenger, and can be monitored in real-time on a custom-built multichannel perfusion system, allowing complete exchange of the solution in the cell chamber. Compared to many omics approaches, Ca^2+^ monitoring has a considerable advantage in its ability to detect instant and transient signaling that will turn on the downstream pathways with different kinetics, which can often be missed by out-of-pace cell harvest or preparations.

Although they are difficult to optimize, we successfully set up monitoring systems with smooth muscle cells and keratinocytes. DPM induced similar Ca^2+^ influx in both cells, slightly deviated by the distinct physiology of each. These cells are present during the interaction between air pollutants and the human body; thus, identifying the same signal suggests highly significant and conserved responses of two different tissues. Moreover, PAE, a traditional medicine known to have potential anti-inflammatory efficacy, could play a role in protecting cells by directly working at the level of signal generation in both cell types. The transient Ca^2+^ influx was mediated by PAR-2, which was confirmed by an agonist that could mimic DPM effect, and PAE’s effect was recapitulated with an antagonist. We also demonstrated that PAR-2 expression can be regulated by DPM, which was reversed by PAE’s protective effect. These results altogether address the notion that PAR-2-dependent Ca^2+^ influx is one of the primary signals that DPM can evoke on the cell surface, and that PAE can directly modulate PAR-2 as well as its expression under stressful conditions. In addition to this, we purified and identified FQA as an active compound of PAE that inhibits Ca^2+^ influx by DPM and regulates the expression of PAR-2. On the other hand, PAE could not completely inhibit DPM-induced Ca^2+^ influx, nor the PAR-2 antagonist. More research is necessary to fully understand the nature of this Ca^2+^ signaling, and a focus on the known transporters and regulators is recommended.

Interestingly, PAE and FQA were able to reverse the upregulation of PAR-2 expression by DPM. We demonstrated that both the transcriptional and the translational regulations exist. However, the change in protein level was more obvious than that in mRNA, especially with higher doses (Figure 4 and Figure 7, and Figure S7). These gaps may have been made by additional factors affecting translational efficiency or post-translational control, which we could not elucidate.

The correlation between skin disorders and air pollution has been suggested by many researchers [32]. Here, we addressed the way by which DPM-induced signaling could lead to damage conditions such as inflammation and skin barrier dysregulation. Proinflammatory cytokines IL-8, IL-6, and TNF-α were induced following Ca^2+^ influx mediated by PAR-2. Furthermore, desmosomal and tight-junction components ZO-1 and Occludin were downregulated. These effects may indicate severe skin damage conditions, where inflammatory responses accelerate cell mobility and immune reactions, and, at the same time, skin barrier integrity is affected. Ultimately, the loss of skin moisturization can lead to severe dermatitis conditions, which have already been reported to be associated with air pollution.

PAE has previously been well studied for its anti-inflammatory effects under similar conditions. It was reported to be effective in lowering TNF-α and other indices of arthritis in mice [33]. Another in vivo study on atopic dermatitis conditions revealed that PAE, with *Sanguisorba officinalis*, could regulate proinflammatory cytokines and the number of immune cells involved in the lesion [34]. Our results corelate with these known effects and suggest underlying molecular mechanisms and a consolidating skin protection effect with potential skin barrier enhancement. The involvement of PAR-2 in DPM-induced skin damage and PAE’s protection may also help future research in tackling questions regarding oxidative damage or hyperpigmentation.

Due to the protective effect of PAE and its active compound, FQA, it could be employed as an active ingredient for dietary or cosmetical formulations. With its long history of herbal remedies in East Asia, daily supplement of PAE could provide protection against respiratory tracts, and topical application of PAE with a proper formula could provide protection to sensitive skin in the area affected by air pollution. More research on the stability of FQA and the safety of PAE use will be helpful to further examine potential industrial applications.

## 5. Conclusions

DPM, as a model of PM2.5, induced transient Ca^2+^ influx in both airway smooth muscle and skin cells, mediated by PAR-2. The signal could lead to an increase in proinflammatory cytokines and a decrease in skin barrier proteins, indicating potent skin damage status. As PAE, or its active compound FQA alone, can inhibit Ca^2+^ influx and the following damage pathways, they represent promising dietary and cosmetical active ingredients.

## Figures and Tables

**Table 1:** Primer sequences of genes analyzed by quantitative real-time PCR.

Gene	Primer Sequence (5′ to 3′)		References
PAR-2	Forward	CTGTGGGTCTTTCTTTTCCGAA	[19]
	Reverse	CAAGGGGAACCAGATGACAGA	
IL-6	Forward	AGTCCTGATCCAGTTCCTGC	[20]
	Reverse	AAGCTGCGCAGAATGAGATG	
IL-8	Forward	TGAGCATCTACGGTTTGCTG	[21]
	Reverse	TGCTTGTCTGGAACAACTGC	
TNF0-α	Forward	GAGGCCAAGCCCTGGTATG	[22]
	Reverse	CGGGCCGATTGATCTCAGC	
β-actin	Forward	CCTCGCCTTTGCCGATCC	[23]
	Reverse	CGCGGCGATATCATCATCC	

## Data Availability

Data is contained within the article or supplementary material.

## Supplementary Materials

The following are available online at https://www.mdpi.com/2218-273X/11/1/23/s1, Figure S1: Dose tests. Figure S2: Western blots of PAR 2. Figure S3: Western blot of Occludin. Figure S4: Western blot of ZO-1. Figure S5: NMR spectra analyses of isolated compounds from PAE. Figure S6: Dose-dependent effect of FQA on DPM stress. Figure S7: Transcriptional regulation of PAR-2 expression by FQA.
