# Supplementary material for: Phellodendron amurense Extract Protects Human Keratinocytes from PM2.5-Induced Inflammation via PAR-2 Signaling
Source: Biomolecules. 2020 Dec 28;11(1):23. doi: 10.3390/biom11010023 (PMC7824043; doi:10.3390/biom11010023)
Supplement: Supplementary file 1 [file biomolecules-11-00023-s001.pdf]

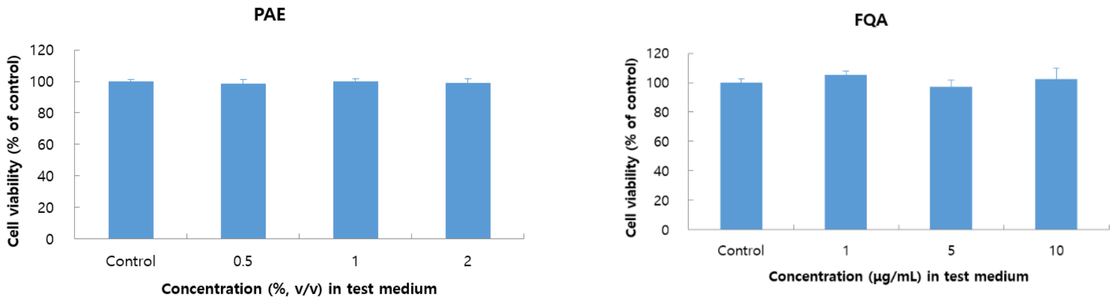


(a) Cytotoxicity test of PAE and its isolated compound, FQA, on HaCaT cells

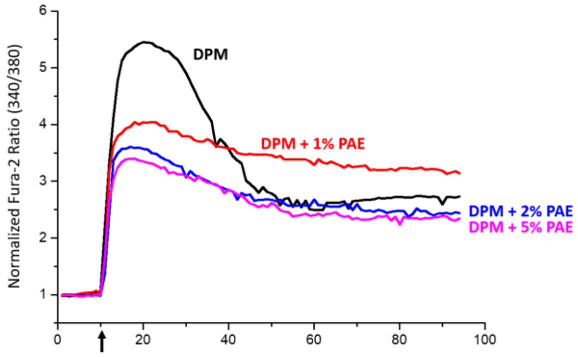


(b) $[Ca^{2+}]_i$ of HBT-SM cells

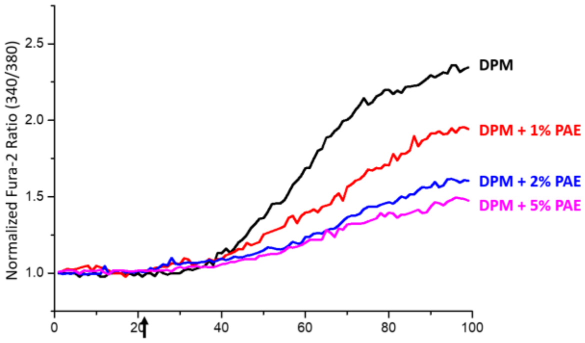


(c) $[Ca^{2+}]_i$ of HaCaT cells

**Figure s1. Dose tests**

As shown here, both PAE and FQA did not show significant cytotoxicity on HaCaT cells, at the effective concentrations used for the biological experiments in the study, and 2% PAE displayed highly significant effect on $Ca^{2+}$ signaling in both HBT-SM and HaCaT cells.

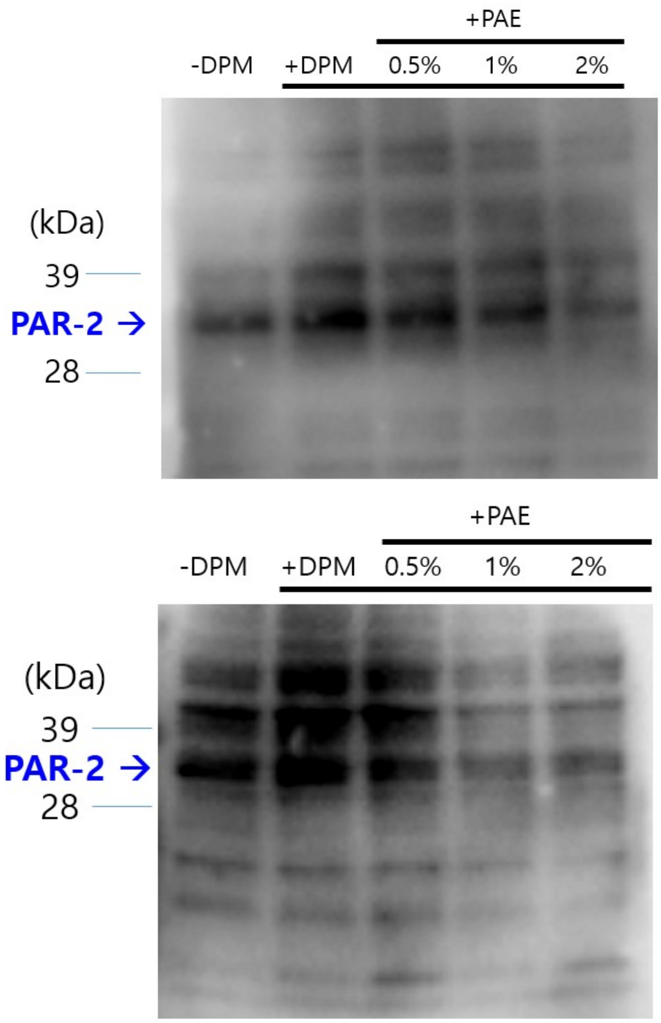


**Figure s2. Western blots of PAR-2**

PAR-2 protein was estimated as 32 kDa, and the strongest signal was detected at the expected size.

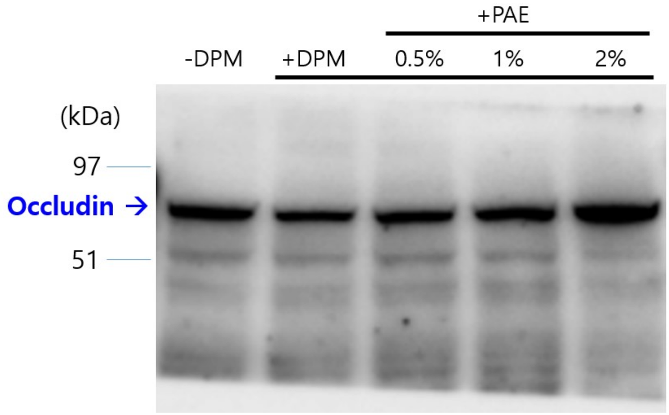


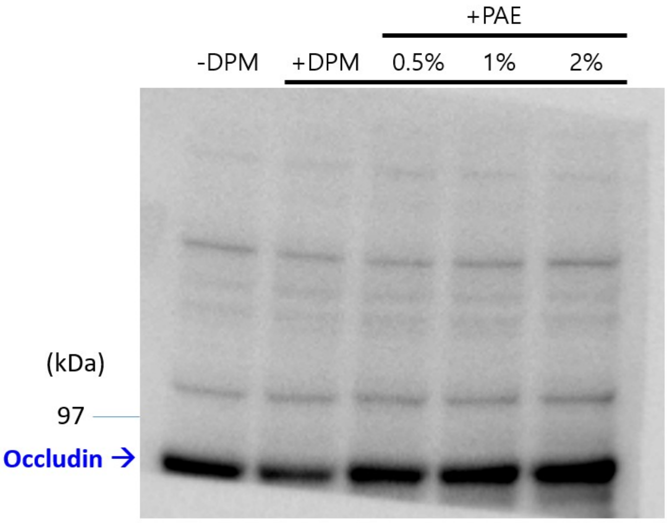


**Figure s3. Western blot of Occludin**

Occludin protein was estimated as 65 kDa, and the strongest signal was detected at the expected size. Extended electrophoresis did not reveal any change of the pattern.

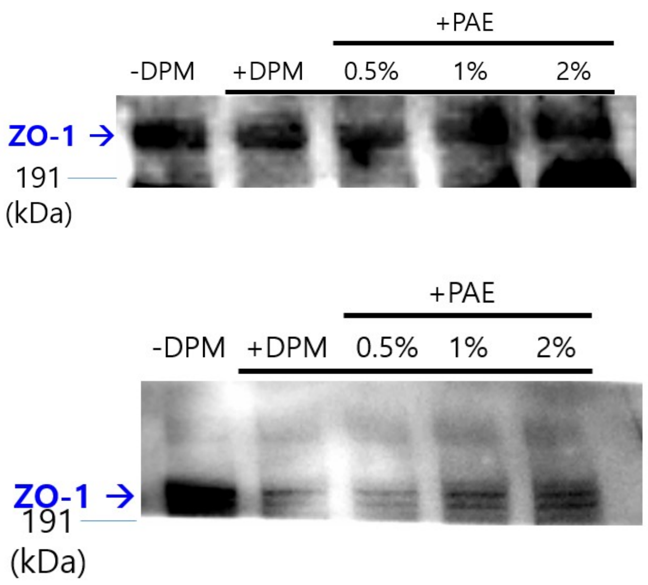


**Figure s4. Western blots of ZO-1**

ZO-1 protein was estimated as 220 kDa, where the signal was detected as known multiple bands. To analyze correctly, a strong nonspecific signal around 180 kDa should be removed by trimming the membrane above the 191-kDa band of pre-stained marker.

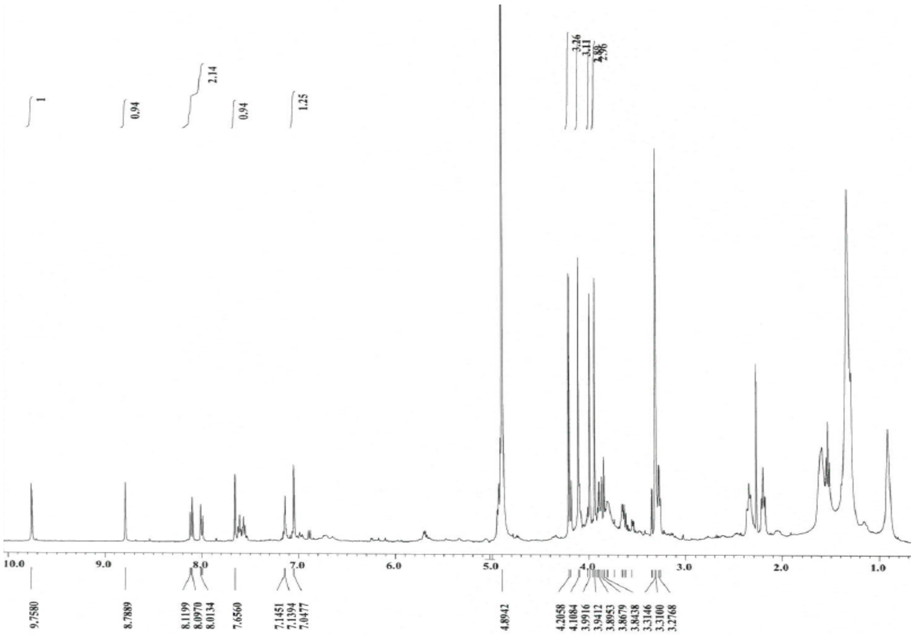

(a) $^{1}$H-NMR Spectra of Palmatine (400㎒, $CD_3OD$)

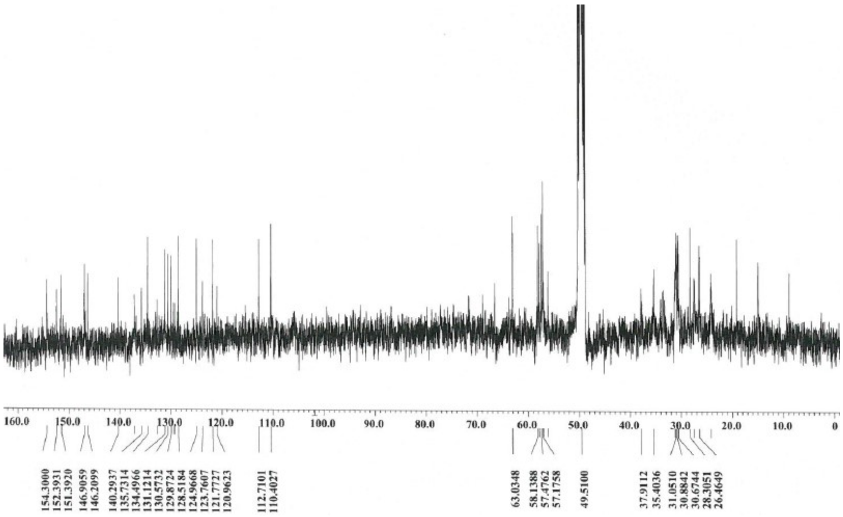

(b) $^{13}$C-NMR Spectra of Palmatine (100㎒, $CD_3OD$)

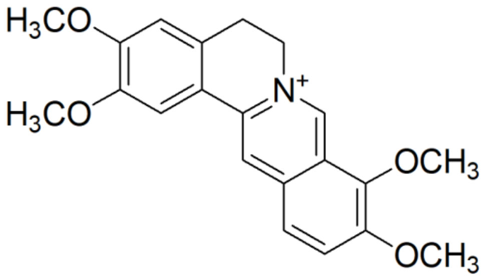


(c) Palmatine

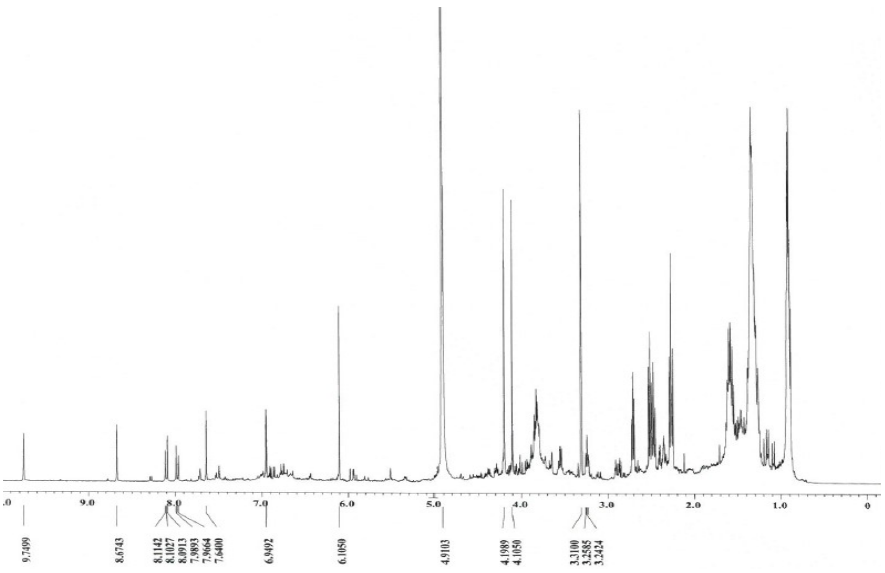


(d) $^1$H-NMR Spectra of Berberine (400㎒, $CD_3OD$)

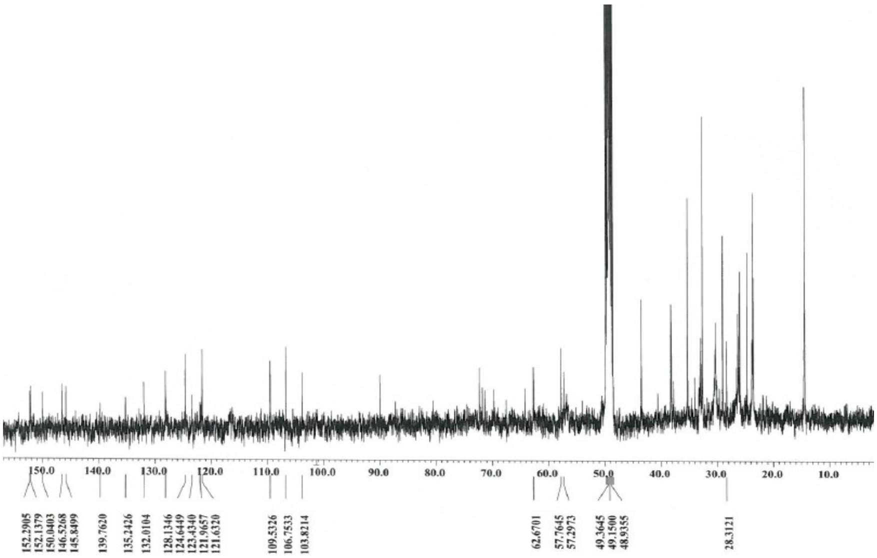


(e) $^{13}$C-NMR Spectra of Berberine (100㎒, $CD_3OD$)

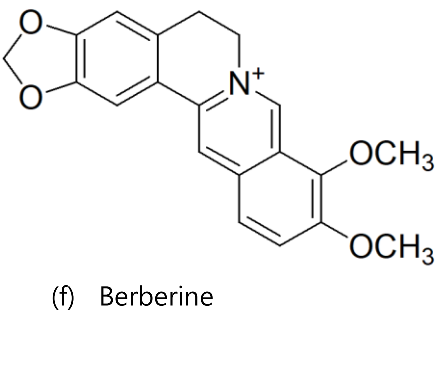


(f) Berberine

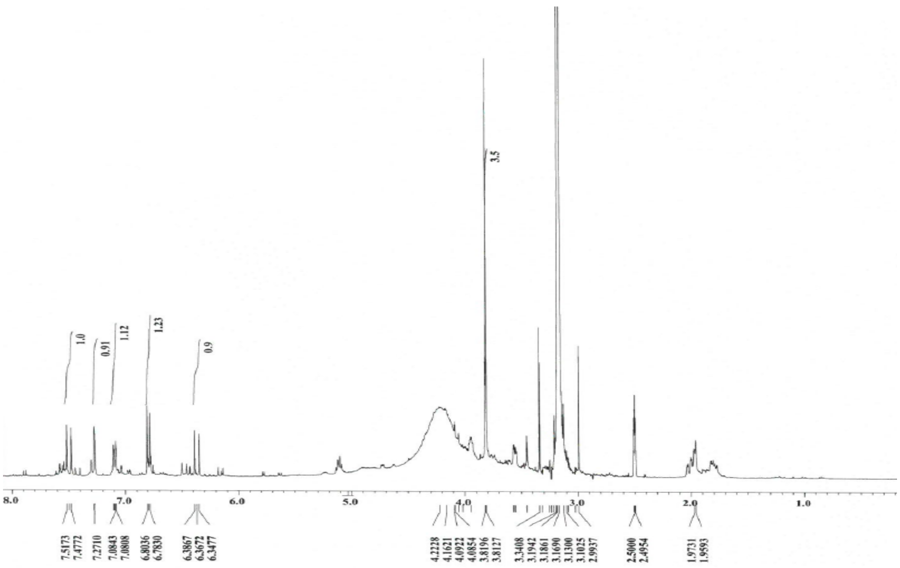


(g) $^{1}$H-NMR Spectra of 4-O-Feruloylquinic acid (400㎒, $CD_3OD$)

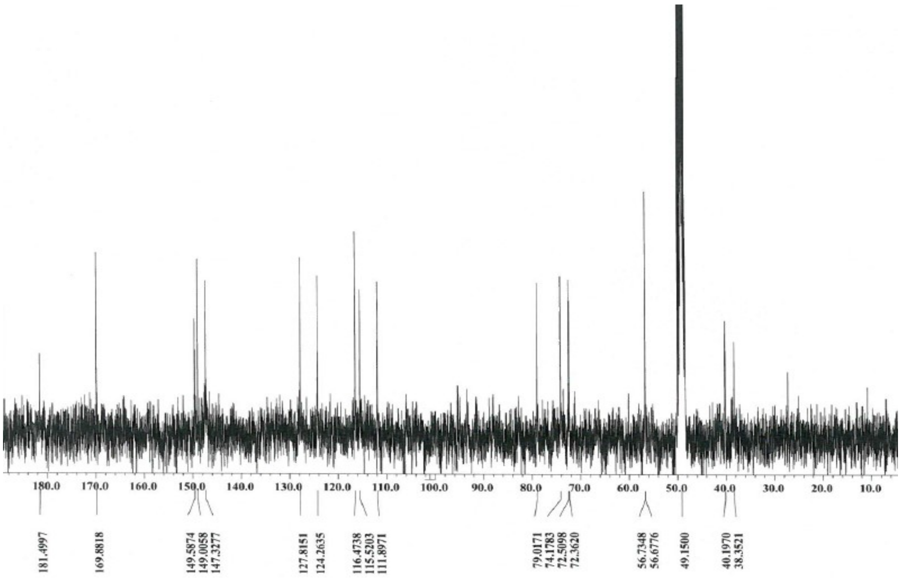


(h) $^{13}$C-NMR Spectra of 4-O-Feruloylquinic acid (100㎒, $CD_3OD$)

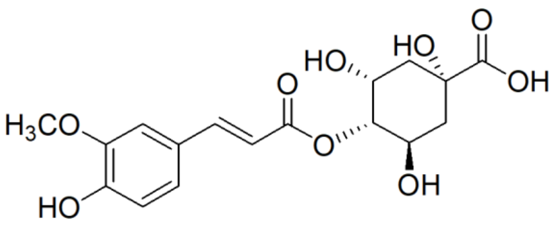


(i) 4-O-Feruloylquinic acid

**Figure s5. NMR spectra analyses of isolated compounds from PAE**

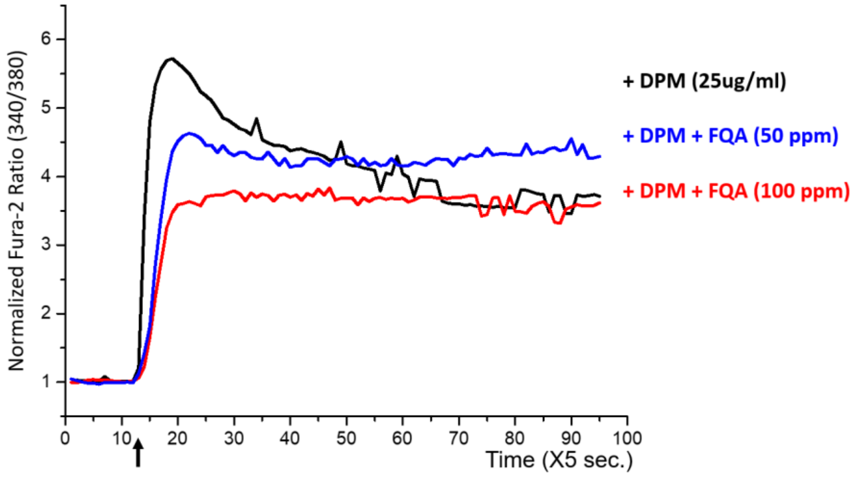


**Figure s6. Dose-dependent effect of FQA on DPM stress**

HBT-SM cells were treated with different concentrations of FQA, in the presence of DPM. 100 ppm showed more significant reduction of $Ca^{2+}$ influx, which was confirmed in HaCaT cells, as Figure 7a.

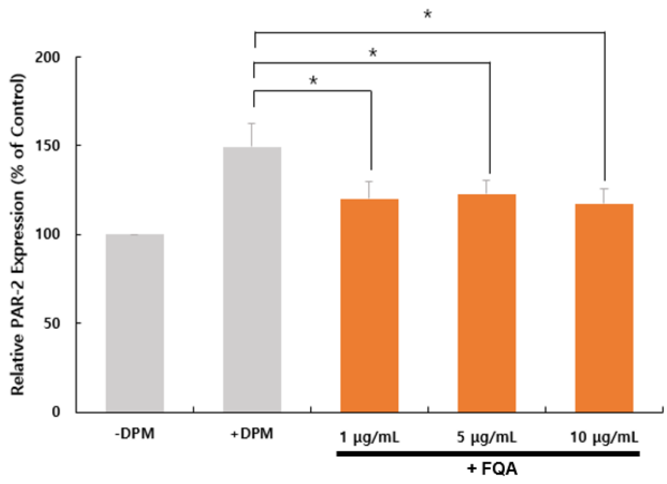


**Figure s7. Transcriptional regulation of PAR-2 expression by FQA.**
